# Different types and numbers metabolic abnormalities and risk of gallbladder stone disease in adults

**DOI:** 10.3389/fnut.2024.1443575

**Published:** 2024-09-09

**Authors:** Tingting Yang, Jianqin Zhong, Renhua Zhang, Fei Xiao, Yuan Wang, Huimin Tao, Feng Hong

**Affiliations:** School of Public Health, the Key Laboratory of Environmental Pollution Monitoring and Disease Control, Ministry of Education, Guizhou Medical University, Guiyang, China

**Keywords:** gallbladder stones, metabolic abnormalities, dysglycemia, dyslipidemia, hypertension, central obesity, abnormal blood uric acid

## Abstract

**Background:**

Metabolic abnormalities in the body increase the risk of gallbladder stones and their complications, which brings a great economic and social burden. The relationship between different types and amounts of metabolic abnormalities and gallstone risk in different sexes is poorly documented and controversial.

**Methods:**

Based on the baseline survey data of the Chinese Multi-Ethnic Cohort (CMEC) study, 4,075 Chinese adults aged 30–79 years with complete abdominal ultrasound results and metabolic index data. Logistic regression model was used to evaluate the correlation between five metabolic abnormalities and gallstones, and to explore the gender difference.

**Results:**

The detection rate of gallbladder stones was found to be 7.0%, with a higher rate in women (8.6%) than in men (4.1%). Logistic results showed adjustment odds ratio (ORs) and 95% confidence interval (95% CI) of dysglycemia + hypertension + central obesity in 3 metabolic combinations was 4.459 (1.653, 12.029). The four metabolic combinations, dysglycemia + dyslipidemia + hypertension + central obesity, dysglycemia + dyslipidemia + hypertension + abnormal blood uric acid and dysglycemia + dyslipidemia + central obesity + abnormal blood uric acid adjusted OR and 95%CI were 3.342 (1.459, 7.659), 5.439 (1.555, 19.018) and 2.971 (1.187, 7.435), respectively. Gender-stratified analysis found that “any three or more metabolic abnormalities and their components were associated with gallstone risk, more significantly in women.

**Conclusion:**

Different types and amounts of five metabolic abnormalities were associated with the risk of gallstone development, and the differences were more significant in women than men.

## Introduction

Gallbladder stone disease (GSD) is the most common subtype of gallstones, which is caused by specific substances in the bile that lead to long-term crystallization of cholesterol into stones (1). Gallbladder stones account for a relatively high proportion of all gallstone diseases, and studies have shown that gallbladder stones account for 76.33% of all gallstone diseases in the Chinese population and 90–95% in Western countries (2). Gallbladder stone disease is the result of the interaction of genetic factors, lifestyle and diet and depends on specific pathogenic mechanisms (1). GSD is one of the major public health problems that not only affects the quality of life of patients but is also associated with a potential risk of cholecystitis, pancreatitis, biliary obstruction and gallbladder cancer, the prevalence of which is expected to increase with the global prevalence of metabolic syndrome (3, 4). The prevalence of gallbladder stone disease has been increasing in recent years in various countries, causing a heavy economic burden due to diagnosis, treatment and indirect medical costs (5–7). Its prevalence is related to the age, lifestyle and dietary habits of the population and may be influenced by race and gender (8–11). GSD has a high prevalence worldwide, which places significant pressure on healthcare resources, including increased need for surgery and hospitalization. In addition, GSD can cause serious complications, such as cholecystitis and pancreatitis, further exacerbating the patient’s health burden and even endangering their lives. Therefore, strengthening prevention strategies and early management measures is of great public health significance to reduce the global health burden of GSD and reduce the pressure on the medical system.

With the in-depth research on the etiology of gallbladder stones, it has been found that many patients with gallbladder stones have abnormal organismal metabolism (12, 13), including hyperglycemia, central obesity, dyslipidemia, and hypertension. Metabolic abnormalities are mainly produced by a combination of environmental and genetic factors, and studies have shown that metabolic abnormalities are associated with the risk of gallbladder stone disease (14, 15). Overweight, obesity, dyslipidemia, insulin resistance and altered cholesterol homeostasis are associated with an increased incidence of gallbladder stones (16). Most of the current studies on the relationship between metabolic abnormalities and gallbladder stone detection have focused on the metabolic syndrome, but the population may have one or more metabolic abnormalities co-existing with the body, and the risk of gallbladder stones with different number and type of metabolic abnormalities is worth to be explored.

In addition, the relationship between different types and amounts of metabolic abnormalities and gallstone risk in different sexes is controversial. A Chinese cohort study showed that as the types and amounts of metabolic abnormalities increased, the risk of gallstone disease increased in men, but not in women. On the contrary, another study suggested that the more metabolic abnormalities present, the higher risk of incident gallstone in both men and women (17, 18).

Therefore, this study aims to discuss the relationship between the number and combination of metabolic abnormalities and the detection of gallbladder stones, and further analyze the effect of different combinations of metabolic abnormalities and the number of metabolic abnormalities on gallbladder stones in different genders, so as to provide a reference for the prevention and control of gallbladder stones.

## Methods

### Study design and participants

**Figure 1:**
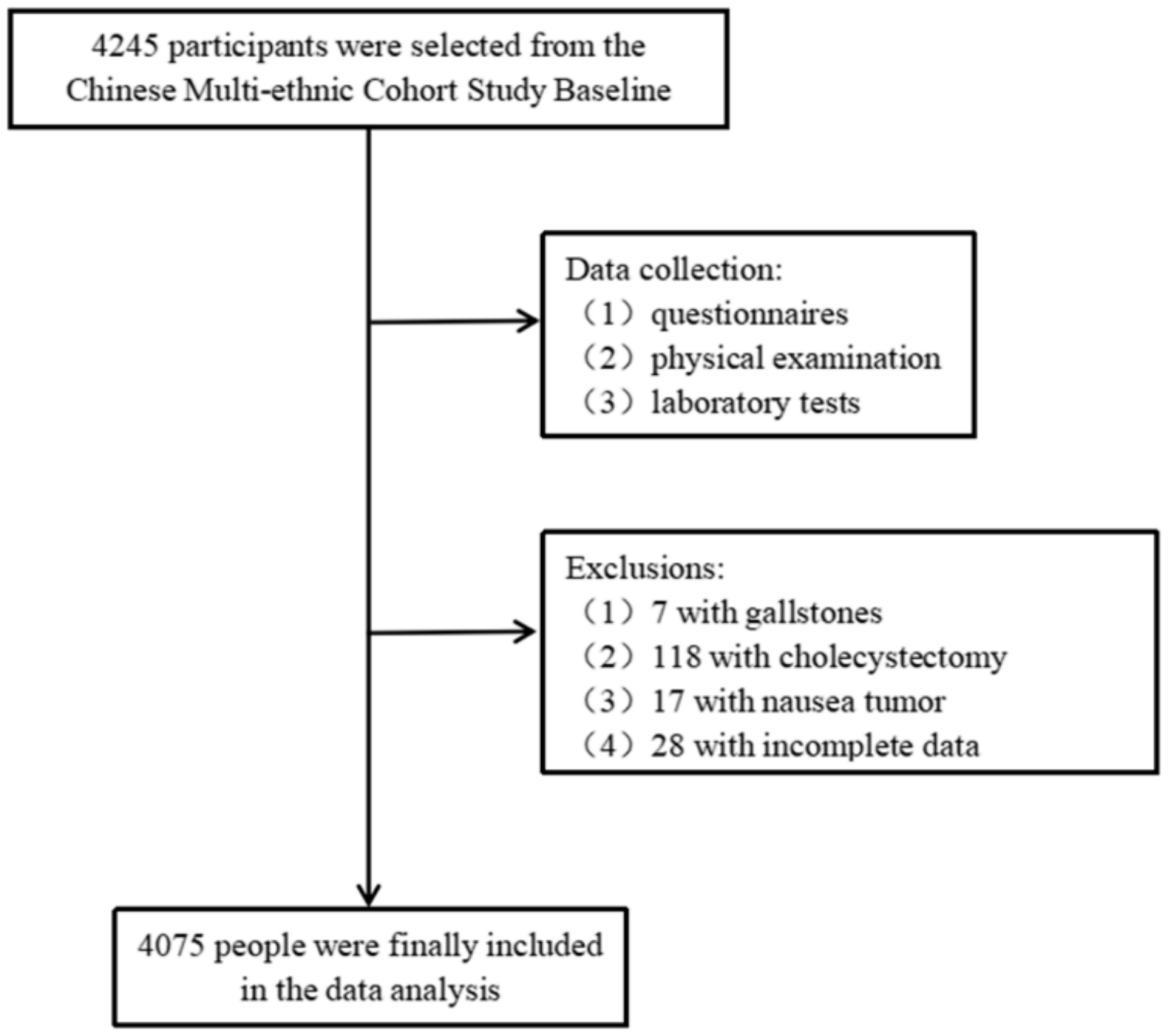
Flow chart of the participant recruitment process.

The data were obtained from the baseline survey database of the China multi-ethnic cohort (CMEC) study, and we selected 4,245 Dong people aged 30–79 years who had attended medical examinations from July 2018 to September 2018 in Qiandongnan Prefecture, Guizhou Province, China, and were required to complete questionnaires, physical examinations, and blood tests. We excluded 7 people with gallstones; 118 people who had previously undergone cholecystectomy; 28 people with missing information on metabolic indexes and abdominal ultrasound results; and 17 people with malignant tumors, and finally included 4,075 study subjects for analysis (Figure 1). The Medical Ethics Review Committee of Sichuan University (K2016038) and the Research Ethics Committee of The Affiliated Hospital of Guizhou Medical University (2018[094]) approved this study. Before participating in the study. All the participants signed the informed consent.

### Assessment of covariates

For more information on the questionnaire and physical examination see elsewhere (19). Demographic characteristics and lifestyle-related factors were collected by uniformly trained professional investigators, and smoking was defined as smoking more than 100 cigarettes in a lifetime (20). Alcohol consumption was defined as drinking alcohol at least once a week for 12 consecutive months (21). In the total population and male population, the adjusted confounders mainly included gender, age, smoking, drinking and region. In the female population, the confounders were adjusted for gender, age, smoking, drinking, region, menopause, birth control pills and breast lump/tumor removal surgery.

### Definition of metabolic abnormalities

Waist circumference (WC) was measured by having the subject stand upright with the feet 25–30 cm apart and a tape measure placed at the end of the line connecting the upper edge of the hip bone and the lower edge of the twelfth rib being measured, and wrapped around the abdomen in a horizontal direction, with results accurate to 0.1 cm. blood pressure was measured using an Omron electronic sphygmomanometer (HEM-7136, Kyoto, Japan) and measured three times after resting for at least 5 min before measurement and averaged. Venous blood was collected after fasting for more than 8 h and sent to Guizhou Jinyu Medical Testing Center Co., Ltd. through cold chain on the same day. Fully automatic biochemical analyzer (Hitachi 7180, Tokyo, Japan) was used to detect blood uric acid (uricase method), blood lipid indexes (cholesterol oxidase method to determine total cholesterol (TC), enzymatic method to determine triglycerides (TG), direct method to determine high-density lipoprotein cholesterol (HDL-C) and low-density lipoprotein cholesterol (LDL-C), fasting plasma glucose (FPG) [hexokinase method]).

(1) Abnormal glucose metabolism: referring to the Chinese Guidelines for the Prevention and Treatment of Type 2 Diabetes (2020 edition) there are diagnostic criteria for hyperglycemia, FBG ≥ 6.1 mmol/L and/or those who have been diagnosed with diabetes (22). (2) Abnormal lipid metabolism: TG ≥ 2.26 mmol/L; HDL-C < 1.04 mmol/L; TC ≥ 6.22 mmol/L; LDL-C ≥ 4.14 mmol/L, with one of the above or diagnosed at a district/county hospital or higher, and currently taking lipid-regulating drugs, as abnormal lipid metabolism (23). (3) Abnormal blood uric acid metabolism: the generally quoted levels are >7 mg/dL (420 μmol/L) for men and >6 mg/dL (360 μmol/L) for women, or those who have been clinically diagnosed with hyperuricemia (24). (4) Hypertension: mean SBP ≥ 140 mmHg and mean DBP ≥ 90 mmHg, or diagnosed hypertension or on antihypertensive medication (25). (5) Central obesity: WC ≥ 90 cm in men and ≥ 85 cm in women was considered central obesity (26).

### Diagnostic criteria for gallbladder stones

By professional/licensed clinicians according to the diagnostic criteria for gallbladder stones in Clinical Hepatobiliary Medicine, all participants underwent a health examination in the morning on an empty stomach using an ultrasound scanner (all approved by the authority) for abdominal ultrasound, gallbladder stones were presented as specific echogenic foci with distal hypoechoic shadows, gallbladder stones could move with the position of the participant, and ultrasound diagnostic results were clear for those with gallbladder stones (7).

### Statistical analysis

Quantitative data that did not follow normal distribution were described by median and quartile spacing and were analyzed by a nonparametric test. Qualitative data were expressed by composition ratio and analyzed by Chi-squared test. Unconditional dichotomous logistic regression was used to further analyze the association between different metabolic abnormality types and numbers and gallbladder stones and to assess the dominance ratio (OR) and 95% confidence interval (95% CI), and we adjusted for confounders such as gender, age, smoking status, alcohol consumption, and region. A further stratified analysis of gender was performed to assess the association between different metabolic abnormality types or amounts and gallbladder stones. All analyses were performed by SPSS software version 22.0 for windows (IBM, Armonk, NY, United States) and R (Version 4.1.2). A two-sided *p*-value of less than 0.05 indicated that the relationship between the two variables was statistically significant.

## Result

### Patient characteristics at baseline

Of the 4,075 respondents included in this study, 285 were diagnosed with gallbladder stones, with a detection rate of 7.0%, of which 4.1% were detected in men and 8.6% in women, and the detection rate of gallbladder stones was higher in women than in men (*p* < 0.001). Gallbladder stones were more likely to occur in people over 60 years of age and living in urban areas (*p* < 0.001), and those with gallbladder stones had higher levels of Blood sugar, TC, TG, LDL-C, Waistline, SBP, DBP and lower levels of HDL-C compared to those without gallbladder stones (*p* < 0.05) (Table 1).

**Table 1:** Patient characteristics at baseline.

Variable	Total (*N* = 4,075)	Gallstone		*p*-value
		No (*n* = 3,790)	Yes (*n* = 285)	
**Sex, *n* (%)**				<0.001
Male	1,422	1,364 (95.9)	58 (4.1)	
Female	2,653	2,426 (91.4)	227 (8.6)	
**Age, *n* (%)**				
30~	428	415 (97.0)	13 (3.0)	<0.001
40~	1,125	1,067 (94.8)	58 (5.2)	
50~	1,274	1,177 (92.4)	97 (7.6)	
60~	900	815 (90.6)	85 (9.4)	
70 ~ 79	348	316 (90.8)	32 (9.2)	
**Smoking status, *n* (%)**				
No	3,161	2,910 (92.1)	251 (7.9)	<0.001
Yes	736	707 (96.1)	29 (3.9)	
Quit smoking	178	173 (97.2)	5 (2.8)	
**Drinking, *n* (%)**				
No	2,347	2,149 (91.6)	198 (8.4)	<0.001
Yes	1728	1,641 (95.0)	87 (5.0)	
**Region, *n* (%)**				
Rural	3,551	3,324 (93.6)	227 (6.4)	<0.001
Urban	524	466 (88.9)	58 (11.1)	
Blood sugar, mmol/L	4,075	5.3 (5.0–5.6)	5.4 (5.1–5.9)	<0.001
TC, mmol/L	4,075	4.7 (4.1–5.3)	4.9 (4.3–5.6)	0.001
TG, mmol/L	4,075	1.4 (1.0–2.1)	1.8 (1.2–2.5)	<0.001
LDL-C, mmol/L	4,075	2.8 (2.3–3.4)	3.0 (2.4–3.6)	0.002
HDL-C, mmol/L	4,075	1.5 (1.2–1.7)	1.4 (1.1–1.6)	<0.001
Waistline, cm	4,075	82.0 (74.5–89.0)	86.5 (80.0–92.7)	<0.001
SBP, mmHg	4,075	120.7 (109.0–134.7)	123.7 (111.7–136.0)	0.041
DBP, mmHg	4,075	78.7 (72.0–86.7)	80.3 (74.0–87.3)	0.042
Serum uric acid, mg/dL	4,075	323.0 (265.0–397.0)	336.0 (280.0–404.5)	0.061

Data represents median (interquartile range) for non-normally distributed variables, or frequencies *n* (%) for categorical variables. Participant characteristics of each group were compared by Mann–Whitney U-test or Chi-square tests.

TC, total cholesterol; TG, triglycerides; HDL, high-density lipoprotein; LDL, low-density lipoprotein; SBP, systolic blood pressure; DBP, diastolic blood pressure.

### The prevalence of gallstone in different metabolic abnormal population

Among the metabolic abnormalities, 4 metabolic abnormalities had the highest prevalence of gallbladder stones and 0 metabolic abnormalities had the lowest prevalence of gallbladder stones. Among the two metabolic indicators, dysglycemia + hypertension had the highest prevalence of gallbladder stones and dysglycemia + central obesity had the lowest prevalence of gallbladder stones; among the three metabolic indicators, dysglycemia + hypertension + central obesity had the highest prevalence of gallbladder stones and dysglycemia + hypertension + abnormal blood uric acid had the lowest prevalence of gallbladder stones; among the four metabolic indicators, dysglycemia + dyslipidemia + hypertension + abnormal blood uric acid had the highest prevalence of gallbladder stones and dyslipidemia + hypertension + abnormal blood uric acid had the lowest prevalence of gallbladder stones. The prevalence of dysglycemia + dyslipidemia + hypertension + central obesity + abnormal blood uric acid gallbladder stones was the highest, and the prevalence of 5 metabolic abnormalities gallbladder stones was 12.2% (Table 2).

**Table 2:** The prevalence of gallstone in different metabolic abnormal population.

Variable	*N*	Gallstone	
		No (*n* = 3,790)	Yes (*n* = 285)
**Number of metabolic abnormalities**			
0 metabolic abnormalities	1,445	1,389 (96.1)	56 (3.9)
1 metabolic abnormality	1,129	1,060 (93.9)	69 (6.1)
2 metabolic abnormalities	808	737 (91.2)	71 (8.8)
3 metabolic abnormalities	470	416 (88.5)	54 (11.5)
4 metabolic abnormalities	182	152 (83.5)	30 (16.5)
5 metabolic abnormalities	41	36 (87.8)	5 (12.2)
**1 metabolic abnormality**			
Dysglycemia	92	89 (96.7)	3 (3.3)
Dyslipidemia	299	278 (93.0)	21 (7.0)
Hypertension	153	147 (96.1)	6 (3.9)
Central obesity	333	306 (91.9)	27 (8.1)
Abnormal blood uric acid	252	240 (95.2)	12 (4.8)
**2 metabolic abnormalities**			
Dysglycemia + dyslipidemia	36	32 (88.9)	4 (11.1)
Dysglycemia + hypertension	25	22 (88.0)	3 (12.0)
Dysglycemia + central obesity	41	40 (97.6)	1 (2.4)
Dysglycemia + abnormal blood uric acid	24	22 (91.7)	2 (8.3)
Dyslipidemia + hypertension	47	43 (91.5)	4 (8.5)
Dyslipidemia + central obesity	203	182 (89.7)	21 (10.3)
Dyslipidemia + abnormal blood uric acid	123	114 (92.7)	9 (7.3)
Hypertension + central obesity	80	72 (90.0)	8 (10.0)
Hypertension + abnormal blood uric acid	71	66 (93.0)	5 (7.0)
Central obesity + abnormal blood uric acid	158	144 (91.1)	14 (8.9)
**3 metabolic abnormalities**			
Dysglycemia + dyslipidemia + hypertension	14	12 (85.7)	2 (14.3)
Dysglycemia + dyslipidemia + central obesity	41	35 (85.4)	6 (14.6)
Dysglycemia + dyslipidemia + abnormal blood uric acid	35	31 (88.6)	4 (11.4)
Dysglycemia + hypertension + central obesity	21	15 (71.4)	6 (28.6)
Dysglycemia + hypertension + abnormal blood uric acid	14	14 (100.0)	0 (0.0)
Dysglycemia + central obesity + abnormal blood uric acid	22	18 (81.8)	4 (18.2)
Dyslipidemia + hypertension + central obesity	49	41 (83.7)	8 (16.3)
Dyslipidemia + hypertension + abnormal blood uric acid	47	45 (95.7)	2 (4.3)
Dyslipidemia + central obesity + abnormal blood uric acid	166	151 (91.0)	15 (9.0)
Hypertension + central obesity + abnormal blood uric acid	61	54 (88.5)	7 (11.5)
**4 metabolic abnormalities**			
Dysglycemia + dyslipidemia + hypertension + central obesity	34	26 (76.5)	8 (23.5)
Dysglycemia + dyslipidemia + hypertension + abnormal blood uric acid	13	9 (69.2)	4 (30.8)
Dysglycemia + hypertension + central obesity + abnormal blood uric acid	13	10 (76.9)	3 (23.1)
Dysglycemia + dyslipidemia + central obesity + abnormal blood uric acid	34	28 (82.4)	6 (17.6)
Dyslipidemia + hypertension + central obesity + abnormal blood uric acid	88	79 (89.8)	9 (10.2)
**5 metabolic abnormalities**			
Dysglycemia + dyslipidemia + hypertension + central obesity + abnormal blood uric acid	41	36 (87.8)	5 (12.2)

### Relationship between different types and amounts of metabolic abnormalities and gallstones in the general population

After adjustment for confounders, compared with 0 metabolic abnormalities, the adjusted ORs for “any 1 item as abnormal,” “any 2 items as abnormal,” “any 3 items as abnormal,” and “any 4 and 5 as abnormalities had adjusted ORs (95% CI) of 1.550 (1.075, 2.236), 2.257 (1.558, 3.269), 3.034 (2.022, 4.553), 4.642 (2.827, 7.621), and 3.371 (1.230, 9.237). Among the 3 metabolic indicators, the adjusted OR (95% CI) for dysglycemia + hypertension + central obesity was 4.459 (1.653, 12.029); among the 4 metabolic indicators, the adjusted OR (95% CI) for dysglycemia + dyslipidemia + hypertension + central obesity, dysglycemia + dyslipidemia + hypertension + abnormal blood uric acid and dysglycemia + dyslipidemia + central obesity + abnormal blood uric acid were 3.342 (1.459, 7.659), 5.439 (1.555, 19.018), and 2.971 (1.187, 7.435) (Table 3).

**Table 3:** The relationship between different types and numbers of metabolic abnormalities and gallstones in the general population.

Variable	Mode l		Mode 2	
	OR (95%CI)	*p*-value	OR (95%CI)	*p*-value
**Number of metabolic abnormalities**				
0 metabolic abnormalities	Ref		Ref	
1 metabolic abnormality	1.165 (1.125, 2.318)	0.009	1.550 (1.075, 2.236)	0.019
2 metabolic abnormalities	2.389 (1.665, 3.430)	<0.001	2.257 (1.558, 3.269)	<0.001
3 metabolic abnormalities	3.220 (2.181, 4.753)	<0.001	3.034 (2.022, 4.553)	<0.001
4 metabolic abnormalities	4.895 (3.047, 7.864)	<0.001	4.642 (2.827, 7.621)	<0.001
5 metabolic abnormalities	3.445 (1.302, 9.113)	0.013	3.371 (1.230, 9.237)	0.018
**1 metabolic abnormality**				
Dysglycemia	0.442 (0.139, 1.407)	0.167	0.424 (0.132, 1.361)	0.149
Dyslipidemia	1.005 (0.634, 1.593)	0.983	0.987 (0.618, 1.577)	0.957
Hypertension	0.533 (0.233, 1.217)	0.135	0.441 (0.192, 1.014)	0.054
Central obesity	1.192 (0.788, 1.801)	0.406	1.160 (0.761, 1.769)	0.491
Abnormal blood uric acid	0.650 (0.359, 1.176)	0.155	0.786 (0.431, 1.436)	0.434
**2 metabolic abnormalities**				
Dysglycemia + dyslipidemia	1.672 (0.587, 4.760)	0.336	1.711 (0.590, 4.958)	0.323
Dysglycemia + hypertension	1.822 (0.542, 6.124)	0.332	1.393 (0.401, 4.835)	0.602
Dysglycemia + central obesity	0.330 (0.045, 2.410)	0.275	0.287 (0.039, 2.107)	0.220
Dysglycemia + abnormal blood uric acid	1.210 (0.283, 5.173)	0.797	1.379 (0.310, 6.140)	0.673
Dyslipidemia + hypertension	1.240 (0.442, 3.480)	0.682	1.096 (0.385, 3.118)	0.864
Dyslipidemia + central obesity	1.577 (0.987, 2.520)	0.057	1.579 (0.979, 2.548)	0.061
Dyslipidemia + abnormal blood uric acid	1.051 (0.528, 2.095)	0.887	1.270 (0.627, 2.573)	0.507
Hypertension + central obesity	1.491 (0.711, 3.128)	0.290	1.165 (0.550, 2.468)	0.690
Hypertension + abnormal blood uric acid	1.008 (0.403, 2.521)	0.987	1.084 (0.422, 2.783)	0.867
Central obesity + abnormal blood uric acid	1.308 (0.745, 2.295)	0.349	1.128 (0.636, 2.003)	0.680
**3 metabolic abnormalities**				
Dysglycemia + dyslipidemia + hypertension	2.225 (0.496, 9.990)	0.297	2.065 (0.444, 9.610)	0.355
Dysglycemia + dyslipidemia + central obesity	2.307 (0.962, 5.532)	0.061	1.881 (0.760, 4.653)	0.172
Dysglycemia + dyslipidemia + abnormal blood uric acid	1.726 (0.605, 4.924)	0.307	1.833 (0.616, 5.455)	0.276
Dysglycemia + hypertension + central obesity	5.412 (2.084, 14.058)	0.001	4.459 (1.653, 12.029)	0.003
Dysglycemia + central obesity + abnormal blood uric acid	2.983 (1.003, 8.8774)	0.049	2.243 (0.731, 6.880)	0.158
Dyslipidemia + hypertension + central obesity	2.641 (1.226, 5.688)	0.013	2.152 (0.987, 4.692)	0.054
Dyslipidemia + hypertension + abnormal blood uric acid	0.588 (0.142, 2.437)	0.454	0.569 (0.135, 2.403)	0.443
Dyslipidemia + central obesity + abnormal blood uric acid	1.339 (0.776, 2.309)	0.294	1.451 (0.831, 2.536)	0.191
Hypertension + central obesity + abnormal blood uric acid	1.742 (0.785, 3.864)	0.172	1.314 (0.582, 2.966)	0.510
**4 metabolic abnormalities**				
Dysglycemia + dyslipidemia + hypertension + central obesity	4.181 (1.875, 9.322)	<0.001	3.342 (1.459, 7.659)	0.004
Dysglycemia + dyslipidemia + hypertension + abnormal blood uric acid	5.980 (1.875, 9.322)	0.003	5.439 (1.555, 19.018)	0.008
Dysglycemia + hypertension + central obesity + abnormal blood uric acid	4.021 (1.100, 14.694)	0.035	3.329 (0.823, 13.465)	0.092
Dysglycemia + dyslipidemia + central obesity + abnormal blood uric acid	2.889 (1.186, 7.037)	0.019	2.971 (1.187, 7.435)	0.020
Dyslipidemia + hypertension + central obesity + abnormal blood uric acid	1.532 (0.760, 3.086)	0.233	1.474 (0.720, 3.019)	0.289
**5 metabolic abnormalities**				
Dysglycemia + dyslipidemia + hypertension + central obesity + abnormal blood uric acid	1.862 (0.725, 4.783)	0.196	1.742 (0.657, 4.621)	0.265

Model 1 is not adjusted. Model 2 is adjusted for gender, age, smoking, drinking and region. OR (95%C), odds ratio (95% confidence intervals).

### Relationship between different types and numbers of metabolic abnormalities and gallstones in the male

**Figure 2:**
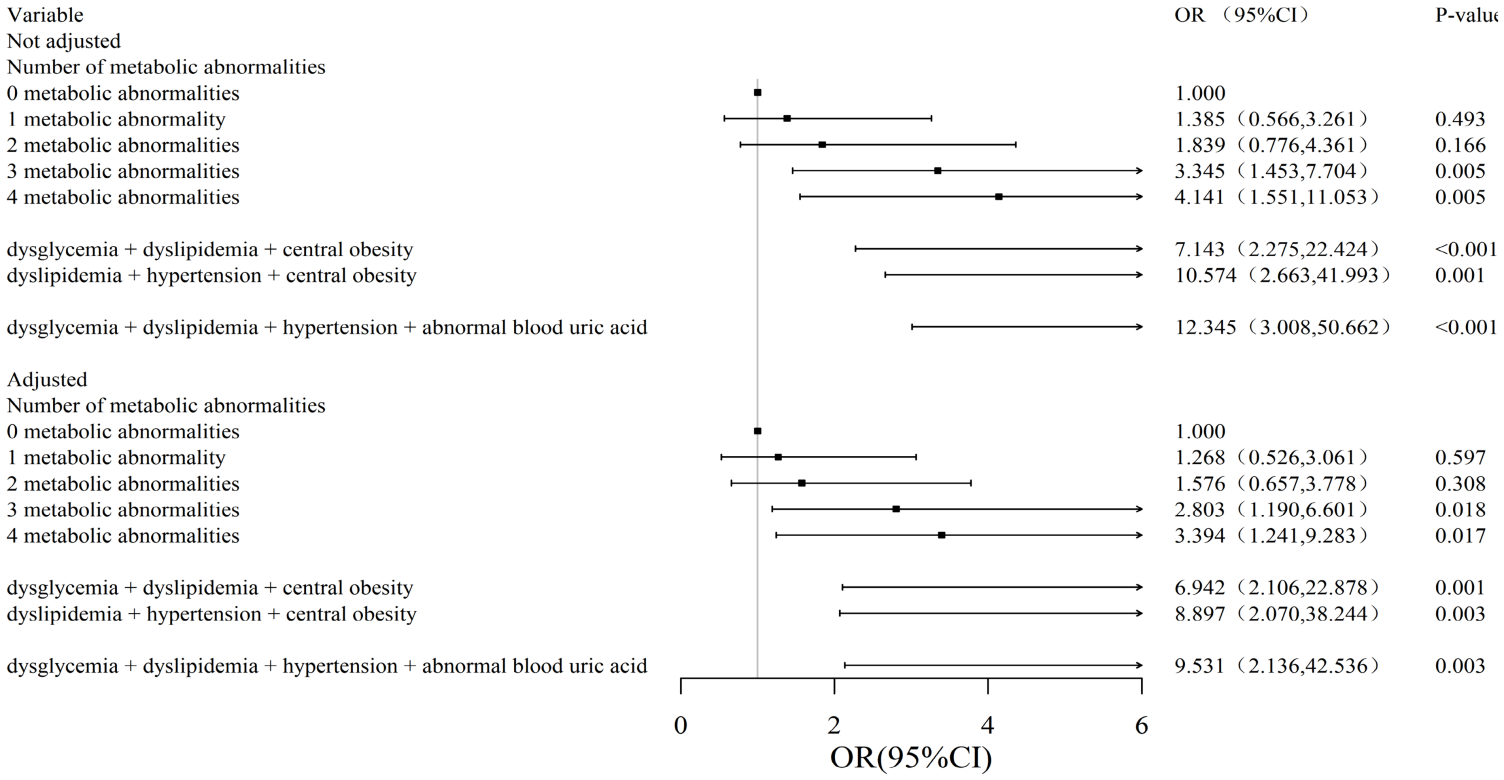
Association between metabolic abnormalities and risk of gallstones in men. Model 1 is not adjusted, model 2 is adjusted for gender, age, smoking, drinking and region. OR (95%CI), odds ratio (95% confidence intervals).

After adjustment for confounding factors, the adjusted ORs (95% CI) for “any 3 abnormalities” and “any 4 abnormalities” were 2.803 (1.190, 6.601) and 3.394 (1.241, 9.283), compared with 0 metabolic abnormalities. The results of the association of abnormal metabolic combinations in males were not found in the female population, while the results of the association of abnormal metabolic combinations in females were as follows: among the 3 metabolic indicators, dysglycemia + dyslipidemia + central obesity and dyslipidemia + hypertension + central obesity adjusted ORs (95% CI) were 6.942 (2.106, 22.878) and 8.897 (2.070, 38.244), respectively; among the 4 metabolic indicators, dysglycemia + dyslipidemia + hypertension + abnormal blood uric acid adjusted OR (95% CI) was 9.531 (2.136, 42.536) (Figure 2).

### Relationship between different types and numbers of metabolic abnormalities and gallstones in the female

**Figure 3:**
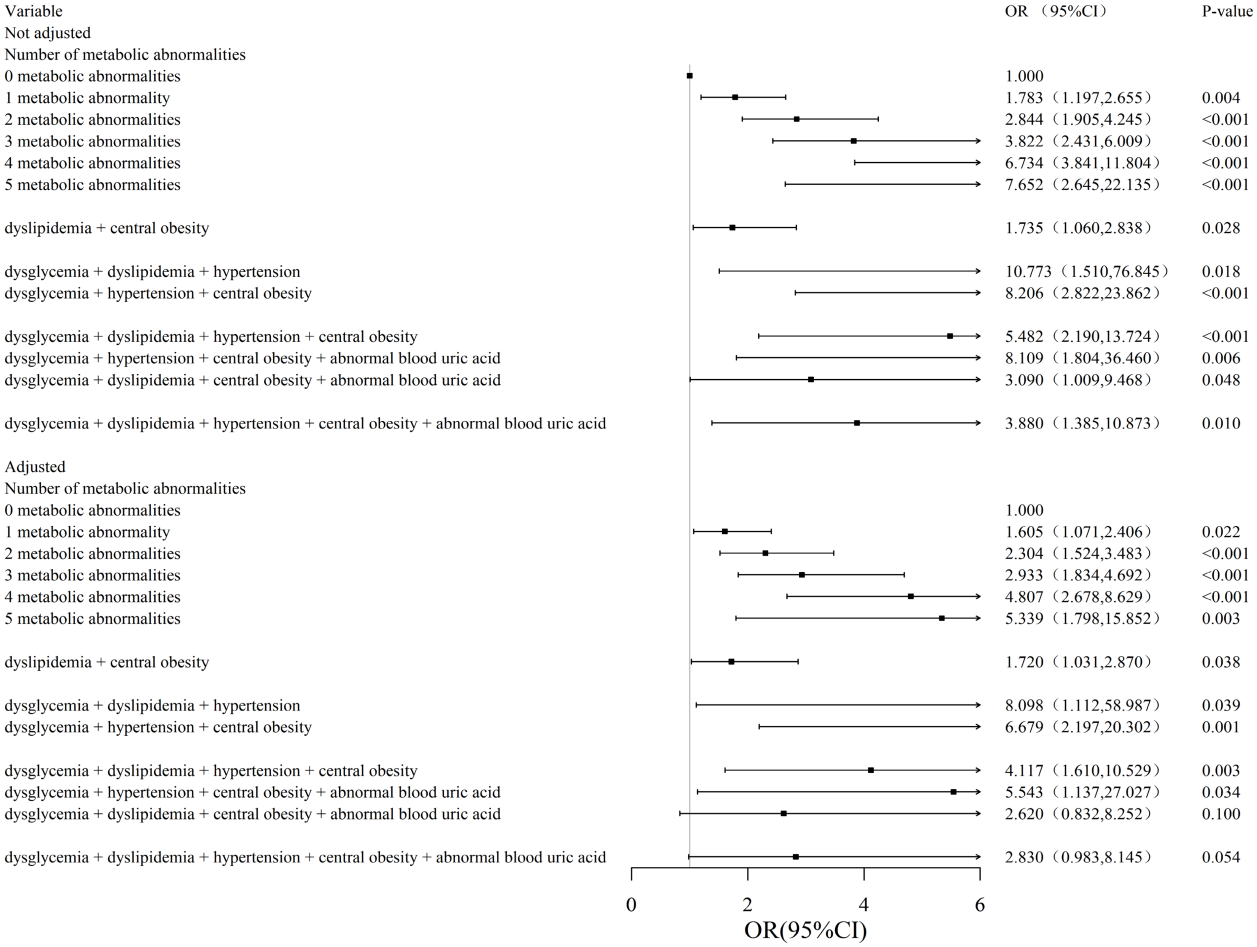
Association between metabolic abnormalities and risk of gallstones in the female. Model 1 is not adjusted, model 2 is adjusted for gender, age, smoking, drinking, region, menopause, birth control pills and breast lump/tumor removal surgery. OR (95%CI), odds ratio (95% confidence intervals).

After adjustment for confounders, compared to 0 metabolic abnormalities, the adjusted ORs (95% CI) for “any 1 item as abnormal,” “any 2 items as abnormal,” “any 3 items as abnormal “, “any 4 and 5 items as abnormalities had adjusted ORs (95% CI) of 1.605 (1.071, 2.406), 2.304 (1.524, 3.483), 2.933 (1.834, 4.692), 4.807 (2.678, 8.629), respectively 5.339 (1.798, 15.852) suggesting that the risk of gallbladder stone detection increases with the number of metabolic abnormalities. The results of the association between the combination of metabolic abnormalities and gallstones in the male population were not found in the female population, while the results of the association between the combination of metabolic abnormalities in the female population were as follows: among the 2 metabolic indicators, the dyslipidemia + central obesity adjusted OR (95% CI) was 1.720 (1.031, 2.870); among the 3 metabolic indicators, the dysglycemia + dyslipidemia + hypertension and dysglycemia + hypertension + central obesity adjusted OR (95% CI) were 8.098 (1.112, 58.987), 6.679 (2.197, 20.302); among the four metabolic indicators, dysglycemia + dyslipidemia + hypertension + central obesity and dysglycemia + hypertension + central obesity + abnormal blood uric acid adjusted OR (95% CI) were 4.117 (1.610, 10.529), 5.543 (1.137, 27.027) (Figure 3).

## Discussion

In the present study, the detection rate of gallbladder stones was found to be 7% in the 30–79 population in China, and the detection rate was significantly higher in women (8.6%) than in men (4.1%), similar to previous reports in the literature (27), considering that it may be because hormones such as estrogen and progesterone play an important role in women-specific high-risk diseases, with estrogen leading to cholesterol supersaturation by increasing cholesterol secretion, and progesterone inhibiting gallbladder contraction and leads to cholestasis (28, 29). However, in contrast to the recent study by Lin et al. (4) and Zhu et al. (9) showing inconsistent results, it is possible that inconsistencies in economic status and lifestyle make the prevalence of gallbladder stone disease in China vary considerably across geographic regions.

We observed a significant correlation between the number of different metabolic abnormalities and the risk of gallbladder stone prevalence in the total population, and different combinations of metabolic abnormalities dysglycemia + hypertension + central obesity, dysglycemia + dyslipidemia + hypertension + central obesity, dysglycemia + dyslipidemia + hypertension + abnormal blood uric acid and dysglycemia + dyslipidemia + central obesity + abnormal blood uric acid were associated with the risk of gallbladder stone prevalence. Chen et al. found that gallbladder stone formation increases with age and that older adults are chronically exposed to multiple chronic factors such as hyperlipidemia, hypertension and diabetes mellitus (DM), which may exhibit decreased gallbladder motility, leading to GSD (30). Abnormal blood glucose may increase the prevalence of gallbladder stones, and diabetes has been shown to promote gallbladder stone formation in ethnic minorities in Xinjiang, and other reports have shown a positive association between diabetes and gallbladder stone formation (8, 31, 32). Fasting blood glucose is elevated due to insulin resistance or a relative decrease in insulin secretion. Insulin resistance increases cholesterol secretion, decreases bile acid synthesis, decreases cholecystokinin response, and slows gallbladder motility, leading to cholesterol retention in the gallbladder, thereby increasing the risk of gallbladder stone formation (33, 34). Elevated blood glucose levels also inhibit gallbladder contraction and bile secretion, promoting gallbladder stone formation (1). High systolic blood pressure is associated with an increased prevalence of gallbladder stones, and although there is no clear mechanism, this association could be explained by the observed increase in insulin resistance in hypertensive patients (35). Patients with hypertension may also have more sympathetic activity, which tends to inhibit intestinal motility and contribute to gallbladder stones (36). Some studies also showed that metabolic indicators of total cholesterol, triglycerides, LDL-C and HDL-C have different effects on gallbladder stones (12, 37). Triglycerides decrease the sensitivity of cholecystokinin, leading to cholesterol accumulation and crystal formation, and may increase bile cholesterol saturation and bile viscosity by increasing mucin production, and elevated cholesterol induces the expression of inflammatory factors, which can lead to cholecystitis and induce gallstone formation (14). Previous studies reported that low HDL-C is a risk factor for GSD (38), that biliary cholesterol is mainly derived from HDL-C, and that insulin resistance is associated with low serum HDL-C concentrations, which may lead to a high catabolic rate of HDL-C particles and thus increase the rate of biliary cholesterol secretion (4, 39). The role of central obesity in gallbladder stone formation may be related to the activity of rate-limiting enzymes, which increase cholesterol synthesis in the liver and promote cholesterol supersaturation and secretion into the bile ducts, thereby inhibiting bile duct peristalsis and leading to gallbladder stone formation (30, 40). Obesity may lead to elevated levels of hepatic cholesterol secretion and supersaturation of biliary cholesterol secretion. Also, cholesterol production is associated with increased body fat (41), biliary cholesterol saturation, bile acid synthesis, turnover rate, and bile acid pool size in obese patients (42). In terms of blood uric acid abnormalities, there is no mechanistic explanation for the relationship between blood uric acid and gallbladder stone formation, Wei et al. found that high level of blood uric acid is an independent risk factor for new-onset gallbladder stone (43). We speculate that gut microbiota may be an important factor. Studies have shown that fecal microbiome composition differed significantly between HUA patients and healthy subjects, gut microbiota could reduce the occurrence and development of HUA by promoting the catabolism of uric acid and reducing the absorption of uric acid in the intestine (44). Moreover, the potential effect of gut microbiota on the pathogenesis of gallbladder stone cannot be ignored, and its disorder can lead to the occurrence of gallbladder stone (45, 46).

In the gender stratification, the number of metabolic abnormalities was found to be three and four associated with gallbladder stones in men, and different combinations of metabolic abnormalities dysglycemia + dyslipidemia + central obesity, dyslipidemia + hypertension + central obesity and dysglycemia + dyslipidemia + hypertension + abnormal blood uric acid were positively associated with the risk of developing gallbladder stones. In the female population, an increase in the number of metabolic abnormalities was found to be associated with the prevalence of gallbladder stones, suggesting that the risk of gallbladder stone detection increases with the increase in the number of metabolic abnormalities. The risk of gallbladder stones was positively correlated with the different metabolic abnormalities of dyslipidemia + central obesity, dysglycemia + dyslipidemia + hypertension, dysglycemia + hypertension + central obesity, dysglycemia + dyslipidemia + hypertension + central obesity and dysglycemia + hypertension + central obesity + abnormal blood uric acid. The risk of gallbladder stones varies by gender and the characteristics of metabolic abnormalities differ by gender (6). Studies have shown that dyslipidemia may be the most important metabolic factor for GSD in men, while in women, women with abdominal obesity are prone to develop GSD, in agreement with our findings (4). In addition, Hsu et al. found that the best obesity indicator for GSD risk may vary by gender, and waist circumference is a better predictor of GSD in women compared to men (47). The different results of the correlation between the number of metabolic abnormalities and different components on gallbladder stones may be due to different regions, sample size, ethnicity, metabolic indicators, and consideration of potential confounding factors in different studies. Based on the results of gender analysis, this study suggests that for the three metabolic abnormalities, men should focus on interventions of dysglycemia + dyslipidemia + central obesity, dyslipidemia + hypertension + central obesity, and women should focus on interventions of dysglycemia + dyslipidemia + hypertension, dysglycemia + hypertension + central obesity; for the four metabolic abnormalities Considering the differences in the physiological mechanisms of men and women, the development of targeted prevention strategies for men and women, respectively, can help prevent the occurrence of gallbladder stones. Interventions such as active prevention of metabolic abnormalities at an early stage, such as lowering blood glucose, blood pressure, blood uric acid, improving dyslipidemia, central obesity, may alleviate the occurrence of gallbladder stones.

The present study has some advantages: firstly, the effect of five metabolic abnormalities individually and in various combinations on gallbladder stones was comprehensively analyzed; secondly, the relationship between the type of metabolic abnormalities and their number and gallbladder stones in different genders was explored by gender; finally, in the female population, we took into account the effect of estrogen and further adjusted for female menopausal status, history of contraceptive pill use, history of breast tumor/lump removal surgery, etc. confounding factors to reduce the bias and minimize potential reverse causality. Of course, there are several limitations of this study: first, this is a cross-sectional survey based on data that cannot determine a causal relationship between metabolic abnormalities and gallbladder stones, and further longitudinal studies are needed to demonstrate this; second, our study did not assess dietary variables associated with gallbladder stones and family history of gallbladder stones; third, although this study controlled for known confounding factors, there may still be confounding factors and information that cannot be controlled, such as ambient air exposure. Considering the above factors, further studies are needed to determine the true relationship between metabolic abnormalities and gallbladder stones. Finally, it is very regrettable that this study did not analyze the association between urine calcium and other related factors and gallstones, which requires further analysis in subsequent studies. The results of this study provide new insights into certain phenomena, but the specific mechanisms still need further investigation. Future studies can explore possible mechanisms to better understand the relationship between metabolic abnormalities and gallstones.

## Conclusion

Overall, our findings suggest that different types of metabolic abnormalities and their number are significantly associated with the risk of developing gallbladder stones, and that the correlation between metabolic abnormalities and gallbladder stones differs by gender. Therefore, better management of metabolic abnormalities, which would help to better understand metabolic abnormalities by gender and to develop gender-specific prevention strategies could help to prevent gallbladder stones, although these results need to be confirmed by more prospective studies in the future.

## Data Availability

The data analyzed in this study is subject to the following licenses/restrictions: data are available from the corresponding author upon reasonable request. Requests to access these datasets should be directed to Feng Hong, fhong@gmc.edu.cn.
